# The role of the orbitofrontal cortex in smoking cue-reactivity in onset phase of smoking behavior, a fMRI study in adolescents

**DOI:** 10.1093/ntr/ntag045

**Published:** 2026-02-23

**Authors:** D R M Tesselaar, A F A Schellekens, J R Homberg, J Booij, C G J Guerrin, J Dieleman, M Luijten

**Affiliations:** Radboud University Medical Center, Department of Psychiatry, 6500 HB, Nijmegen, The Netherlands; Donders Institute, Donders Center of Medical Neurosciences, 6525 EN, Nijmegen, The Netherlands/Radboud University Medical Center, Department of Medical Neuroscience, Nijmegen, The Netherlands; Nijmegen Institute for Scientist-Practitioners in Addiction (NISPA), 6500 HE, Nijmegen, The Netherlands; Radboud University Medical Center, Department of Psychiatry, 6500 HB, Nijmegen, The Netherlands; Donders Institute, Donders Center of Medical Neurosciences, 6525 EN, Nijmegen, The Netherlands/Radboud University Medical Center, Department of Medical Neuroscience, Nijmegen, The Netherlands; Nijmegen Institute for Scientist-Practitioners in Addiction (NISPA), 6500 HE, Nijmegen, The Netherlands; Radboud University Medical Center, Department of Psychiatry, 6500 HB, Nijmegen, The Netherlands; Donders Institute, Donders Center of Medical Neurosciences, 6525 EN, Nijmegen, The Netherlands/Radboud University Medical Center, Department of Medical Neuroscience, Nijmegen, The Netherlands; Amsterdam UMC, University of Amsterdam, Department of Radiology and Nuclear Medicine, 1105 AZ, Amsterdam, The Netherlands; Radboud University Medical Center, Department of Medical Imaging, 6500 HB, Nijmegen, The Netherlands; Radboud University Medical Center, Department of Psychiatry, 6500 HB, Nijmegen, The Netherlands; Donders Institute, Donders Center of Medical Neurosciences, 6525 EN, Nijmegen, The Netherlands/Radboud University Medical Center, Department of Medical Neuroscience, Nijmegen, The Netherlands; GGD Noord- en Oost Gelderland, Warnsveld, 7231 AC, The Netherlands; Behavioural Science Institute, Radboud University, Nijmegen, 6525 XZ, The Netherlands; Behavioural Science Institute, Radboud University, Nijmegen, 6525 XZ, The Netherlands

**Keywords:** smoking, adolescents, smoking cue-reactivity, fMRI, tobacco use disorder

## Abstract

**Introduction:**

Adults with tobacco use disorder show increased activity patterns in frontostriatal regions compared to non-smokers when viewing smoking-related cues. Smoking mostly starts during adolescence. It is important to examine the development of smoking cue-reactivity patterns during early phases of tobacco use, specifically in adolescents who have not yet developed habitual smoking.

**Methods:**

We tested in adolescents whether regular (*n* = 32) and experimental (*n* = 54) smoking adolescents differed from controls (*n* = 47) during an active smoking cue-reactivity task, through functional magnetic resonance imaging (MRI) with a whole brain and region of interest (ROI) approach (orbitofrontal cortex (OFC), dorsolateral prefrontal cortex, middle frontal gyrus and striatum). Additionally, we investigated the association between smoking cue-related activity in significant ROIs and levels of pleasure of smoking and habitual smoking among all smoking adolescents.

**Results:**

Whole-brain analyses showed no differences in smoking cue-reactivity between the groups. Region of interest analyses showed a higher smoking cue-related activity in the left OFC in regular smoking adolescents compared to controls. We found no dose–response relationships between smoking cue-related activity in the OFC and pleasure of smoking or habitual smoking.

**Conclusions:**

Overall, we only observed increased smoking cue-related brain activity in the left OFC in regular smoking adolescents compared to non-smokers in the ROI analyses. These results suggest that OFC reactivity to smoking cues could be one of the first signs of the development of tobacco use disorder.

**Implications:**

The understanding of the neurobiological development of smoking behavior and tobacco use disorder fosters optimal prevention and intervention strategies. Our data show altered neurobiological responses in the orbitofrontal cortex to smoking cues in non-addicted weekly smoking adolescents, suggesting that orbitofrontal cortex reactivity could be one of the first signs of tobacco use disorder development.

## Introduction

Nicotine and tobacco use are leading causes of preventable disease and death worldwide, contributing to 165 million disability adjusted life years, being the second-highest leading risk factor for disease burden, and accounts for 6.18 million deaths per year worldwide.^1^ Smoking commonly starts during adolescence. Adolescents are more vulnerable to the highly addictive properties of nicotine, and given the neurodevelopment during this age period, tobacco use can have long-term impact on brain maturation.^2^ Based on trend levels, the World Health Organization projected that in 2025, roughly 17.1% of adolescents smoke tobacco worldwide.^3^ In 2023, 16.1% of the Dutch adolescents (12-16 years) used tobacco once, and the prevalence of daily smoking among these adolescents increased from 0.1% at 12 years to 4.7% at 16 years.^4^ Insight into the onset phase of nicotine and tobacco use during adolescence is thus key for preventing smoking-related harm.

Cigarette smoking, like other forms of substance use, often starts with experimentation and impulsive behavior, which, for some, comes with experiencing pleasure.^5^^,^^6^ However, long-term use can gradually transform into more habitual or compulsive use driven by the need to alleviate negative emotions, stress, or withdrawal.^7–9^ These behavioral shifts ultimately can result in tobacco use disorder (TUD).^9^^,^^10^ While many studies focus on TUD, more studies need to focus on the early onset phase, as it could help gain insight into neurobiological risk makers associated with the development of TUD.

One well-known characteristic of people with TUD is their altered neural response to smoking-related cues that, through repeated exposure, result in conditioned triggers that induce craving and use of nicotine.^11^ Meta-analyses found that adults with TUD exhibit increased activity in the frontostriatal areas, such as the orbitofrontal cortex (OFC), middle frontal gyrus (MFG), dorsolateral prefrontal cortex (dlPFC), and striatum, when viewing smoking-related cues.^11^^,^^12^ Similar results have been observed in daily smoking adolescents compared to non-smoking adolescents.^13^^,^^14^ While these findings provide insights into cue-reactivity in TUD and daily smoking, studies specifically addressing the onset phase of TUD are lacking.

One approach to study this process is to examine how neural cue-reactivity varies with smoking frequency. No such studies have been conducted in (adolescent) non-addicted smoking people to our knowledge. Nevertheless, similar dose–response effects have been investigated in alcohol use. For example, heavy drinking adults showed increased cue-reactivity in the striatum compared to light drinking adults.^15^ Similarly, there is a difference in the amplitude of event-related cue-reactivity potentials measured with electroencephalogram in light versus heavy non-problematic drinking adults.^16^^,^^17^ A longitudinal study also showed that young adults (18-22 years) who transitioned from moderate to heavy drinking within a year portrayed higher alcohol cue-reactivity at baseline in the ventromedial prefrontal cortex, OFC, caudate nucleus, insula, and pre-central gyrus, compared to groups with continuous moderate or continuous heavy drinking.^18^ Furthermore, they observed a moderate positive correlation between alcohol cue-reactivity at baseline and drinking levels, and alcohol-related problems a year later.^18^ These results suggest that cue-reactivity may reflect not just current use, but also predispositions for the development of substance use disorders (SUDs). Whether similar patterns exist in the onset phase of smoking is currently unknown.

Although behavioral distinctions between experimental and regular smoking adolescents have been observed, such as an increase in impulsive and compulsive use for regular smoking adolescents,^19^ no studies have specifically examined brain activity in response to smoking cues between adolescents with an experimental or regular smoking pattern and adolescents who have never smoked. To address this knowledge gap, we investigated the blood-oxygen level dependence (BOLD)-response during a smoking cue-reactivity task in regular smoking adolescents (at least weekly), experimental smoking adolescents (less than weekly), and non-smoking control adolescents (never smoked). We hypothesized that adolescents with a regular smoking pattern would show stronger frontostriatal activation to smoking-related cues, compared to never-smoking adolescents, while those with an experimental smoking pattern would not differ significantly from either group. Additionally, to explore if these differences can be explained by pleasure of smoking or habitual behavior of smoking, we investigated in both experimental and regular smoking adolescents whether the BOLD-response to smoking-related cues is associated with self-reported levels of pleasure of smoking and habitual smoking.

## Methods

### Preregistration

The current paper uses data of a larger longitudinal study. Preregistration of the current research questions, hypotheses, and analyses can be found at Open Science Framework (https://osf.io/67wa4). Preregistration of the complete project can be found at Open Science Framework (https://osf.io/6jw9p/).

### Participants (procedure and selection)

All participants or their legal representatives (if participants were below the age of 16 years) provided informed consent. The Medical Ethical Committee of Arnhem-Nijmegen approved the study protocol (#2015-2120). From the complete project sample (*n* = 151), participants with missing data for the smoking cue-reactivity task were excluded (*n* = 15), as well as participants who reported to fell asleep during scanning (*n* = 2) or who reported dizziness (*n* = 1). A total of 133 adolescents remained for the current analyses.

The inclusion criterion for the project sample was age between 12 and 19 years, and exclusion criteria were use of psychoactive medication that could not be stopped for 24 hours, functional magnetic resonance imaging (fMRI) contraindications, and history of neurological diseases. An additional exclusion criterion for controls was ever smoked a complete cigarette or more, and an additional baseline inclusion criterion for smoking adolescents was smoking 5-500 cigarettes in their lifetime, but no daily smoking. All participants were asked to abstain from alcohol, but not tobacco, for at least 24 hours before scanning.

The complete project consisted of three data collection waves, with MRI measurements at baseline and 12-month follow-up used in the current study. To create distinct groups for the current analyses, participants were asked to rate their smoking behavior based on 9 statements: (1) I smoke at least once a day; (2) I do not smoke daily, but at least once a week; (3) I do not smoke weekly, but at least once a month; (4) I smoke less than once a month; (5) I try smoking every once in a while; (6) I quit smoking, after I smoked at least once a week; (7) I quit smoking, after I smoked less than once a week; (8) I tried smoking every once in a while, but I quit and (9) I never smoked. All control participants (*n* = 47) only had baseline measurements, and as they never smoked, they did not rate their smoking behavior. The experimental smoking-group consisted of participants who responded positively to statements 3, 4, or 5 at baseline or 12-month follow-up, and the regular smoking-group consisted of participants who responded positively to statement 2 at baseline, or to 1 or 2 at 12-month follow-up. Initially, smoking participants were classified according to follow-up data. If no group could be determined on this basis, or if the MRI was unavailable, baseline data was used. We did not observe any indications for learning effects on smoking cue-related brain activation between measurements. For more details, see supplementary materials (Supplementary Materials 8.1.1).

### Measurements

#### Nicotine dependence

To assess nicotine dependence in the smoking participants, the 6-item Fagerström Test for Nicotine Dependence (FTND) was used.^20^ This questionnaire assesses physical dependence on cigarettes and/or smoking. The fourth item of the questionnaire, “how many cigarettes a day do you smoke?” was determined based on the number of monthly smoked cigarettes. The reported number of cigarettes per day multiplied by 365 divided by 12 for participants who chose statement 1, or number of cigarettes per week multiplied by 52, divided by 12 for regular smoking adolescents, and by the reported number of cigarettes per month for experimental smoking adolescents. A score of 0-2 indicates very low dependence, while a score of 8 or higher indicates high dependence. Internal consistency of this questionnaire was acceptable, as Cronbach’s alpha ranged from 0.45 to 0.83.^21^

#### Modified reasons for smoking scale – pleasure of smoking

The pleasure of smoking subscale of the Dutch version of the Modified Reasons for Smoking Scale – pleasure of smoking scale (MRSS-POS) was used to measure pleasure of smoking.^22^ Participants rated four statements on a scale from never (1) to always (5), for example, “When I smoke a cigarette, part of the enjoyment is watching the smoke as I exhale”. The score was averaged over all four items and could lie between 1 and 5. Internal consistency of this subscale was acceptable, as Cronbach’s alpha for this subscale was 0.70.^22^ Controls did not complete the MRSS-POS.

#### Self-Report Habit Index

The 12-item Self-Report Habit Index (SRHI) questionnaire was used to assess habitual smoking behavior.^23^ Statements were rated on a scale from completely agree (1) to completely disagree (5), for example, “Smoking is something I start doing before I realize I’m doing it”. The score was averaged over all twelve items and could lie between 1 and 5. A lower score meant more habitual behavior, a high score meant little or no habitual behavior. Internal consistency of this questionnaire was acceptable, as Cronbach’s alpha was 0.95.^24^ Controls did not complete the SRHI.

#### Pubertal Development Scale

The participants included in this study were undergoing pubertal developmental changes, thereby increasing variance in brain development across participants.^25^ To control for this, we included the Pubertal Development Scale (PDS) by Petersen et al.^26^ as covariate. This self-reported questionnaire contains questions regarding the status of secondary sexual characteristics (eg, body hair, growth spurt, menstruation cycle, lower voice, etc.) to determine the developmental phase (“1: has not started yet” to “4: seems already finished”). The score was averaged over eight items for females and five items for males, and could lie between 1 and 4. Internal consistency of this questionnaire was acceptable as Cronbach’s alphas were 0.807 and 0.683 for boys and girls, respectively.^26^

#### Smoking cue-reactivity task

This study used an active cue-reactivity task during fMRI scanning. Participants were instructed to press a button as fast as possible when a new stimulus appeared on the screen. This was done to keep participants engaged in the task. Stimuli were presented one by one, in the middle of the screen, and consisted of 32 smoking-related pictures, 32 non-smoking (neutral) related pictures, and 32 romantic pictures. All pictures were matched on visual complexity (eg, brightness and color), the number of people, and the sex distribution of people presented in the picture. The task consisted of 24 blocks, with each block including 4 pictures from the same category. Each picture was shown once between 3 and 5 seconds (ss) (M = 4.00, SD = .56), and each block lasted 16 seconds. The order of the blocks was counterbalanced. After each block, a fixation cross was shown for 6.5-10 seconds (M = 7.94, SD = 1.31). The total task duration was about 12 minutes. Brain activation and reaction time were measured during this task using fMRI. The fMRI analyses of this study focused on the smoking-related and neutral pictures only. Results of the reaction time are reported in supplementary materials (Supplementary Table 1).

### fMRI data acquisition

fMRI data were acquired on a Siemens 3 Tesla Skyra MRI scanner (Siemens Medical System, Erlangen, Germany) using a 32-channel coil. Functional T2*-weighted imaging was obtained using multi-echo echoplanar imaging to acquire 39 axial slices in interleaved ascending order (voxel size 3.5 × 3.5 × 3.0 mm; matrix 64 × 64; repetition time 2.020 ms; echo times 7, 16.3, 26, 35, and 44 ms; flip angle 80°). A high-resolution weighted anatomical scan was also obtained (MPRAGE; 192 axial slices; voxel size 1 × 1 × 1 mm; matrix 256 × 256; repetition time 2.300 ms; echo times 3.03 ms; flip angle 8°).

### Data analysis

#### fMRI data analyses

Functional imaging analyses were performed using SPM12 (www.fil.ion.ucl.ac.uk/spm), with the MarsBar 0.44 toolbox^27^ and WFU Pickatlas version 3 toolbox.^28^^,^^29^ For details regarding fMRI data preprocessing, see Boormans et al.^30^ and Dieleman et al.^31^ Briefly, first, images were converted to National Individual Floating Transport Infrastructure (NIFTI) and realigned using the first TE-images. The 3 first BOLD images were used to create contrast-to-noise ratio maps for each echo. Additional steps included co-registration of anatomical and functional images, normalization into Montreal Neurological Institute (MNI) spaces, and smoothing with a full-width-at-half-maximum of 8 mm. Afterwards, motion artifacts were removed using FMRIB Software Library (FSL) ICA_AROMA (independent component analysis-based automatic removal of motion artifacts).

Our first-level GLM included three regressors, modeling smoking-related stimuli, neutral stimuli, and romantic stimuli. Second-level analyses consisted of two steps. First, we computed the contrast smoking stimuli > neutral stimuli to represent smoking cue-reactivity. This contrast was used for all follow-up analyses. A one-sample *t*-test was performed to show whether the task activated the expected brain networks, using a cluster-level family-wise error (FWE) correction threshold of *P*_FWE_ <.05 (Supplementary Figures 1 and 2, Supplementary Tables 2 and 3).

Secondly, we executed three pairwise *t*-test comparisons of smoking cue-related activity: (1) between controls and experimental smoking adolescents, (2) between controls and regular smoking adolescents, and (3) between experimental and regular smoking adolescents. Results were considered significant if *P*_FWE_ <.05.

As preregistered, whole-brain analyses were followed by region of interest (ROI) analyses. ROIs were defined within frontostriatal areas in line with Lin et al.,^11^ that is, the OFC, MFG, dlPFC, and striatum. Lin et al.^11^ is the most recent meta-analyses investigating the neural substrates of smoking cue-related activity in the brain of individuals with TUD compared to healthy controls. The OFC and MFG were created with Individual Brain Atlases using Statistical Parametric Mapping (IBASPM) 116 labels, the dlPFC was created with Brodmann areas 9 and 46, and the striatum was created with IBASPM 71 labels.^32^ All masks are available upon request. A small cluster correction was executed for each ROI separately. Results were considered significant if *P* <.05.

Finally, we performed linear regression analyses between significant ROIs and (1) pleasure of smoking, and (2) habitual smoking (self-reported habit index). A small cluster correction was executed for each ROI separately. Results were considered significant if *P* <.05.

Age, sex, educational level, and pubertal developmental state score were added as covariates in all fMRI analyses. Educational level was labeled 1 for “practical, which includes MBO (senior secondary vocational education)/VMBO (preparatory secondary vocational education), 2 for ‘theoretical’ which includes HAVO (school of higher general secondary education), and 3 for ‘pre-university’” which includes VWO (pre-university education). All covariates were centered with their overall mean.

Exploratory sensitivity analyses were executed with three additional covariates for number of e-cigarette and cannabis smoking during the last 30 days and glasses of alcohol per week (see Supplementary Materials 8.1.4), as e-cigarette smoking was associated with smoking cue-reactivity.^33^ Additionally, a secondary exploratory analysis was executed with number of cigarettes smoked per month as independent variable for a combined smoking adolescents group.

## Results

### Descriptive statistics

Controls, experimental, and regular smoking adolescents did not differ in sex distribution, education level, PDS score, smoking initiation age, or regular e-cigarette use (*P* >.05). However, controls were significantly younger than regular smoking adolescents (*P* = .011), and drank fewer glasses of alcohol per week than experimental and regular smoking adolescents (*P* <.001). Regular smoking adolescents had a lower self-reported habit index score (*P* <.001), smoked monthly more cigarettes (*P* <.001) and exhibited a significant higher FTND score (*P* =.047) compared to experimental smoking adolescents. Table 1 includes a summary of participants’ demographics.

**Table 1:** Demographics of participants.

	Controls	Experimental smoking	Regular smoking	*P*-value
***N* (% male)**	47 (29.8%)	54 (33.3%)	32 (28.1%)	.864
**Age (years)**	16.6 (1.1)	17.1 (1.3)	17.4 (1.2)	<.01^a^
**SRHI**	-	4.4 (0.5)	3.7 (0.6)	<.001
**MRSS – POS**	-	2.9 (1.0)	3.0 (0.8)	.952
**PDS female** ** male**	3.9 (0.3) 3.2 (0.4)	3.9 (0.3) 3.4 (0.6)	4.0 (0.3) 3.4 (0.6)	.544
**Education level** ** Practical, *n* (%)** ** Theoretical, *n* (%)** ** Pre-university, *n* (%)**	25 (53.2%) 10 (21.3%) 12 (25.5%)	27 (50.0%) 14 (25.9%) 13 (24.1%)	19 (59.4%) 8 (25.0%) 5 (15.6%)	.828
**Monthly smoking**	-	4.4 (5.3) Range: [0-30]	55.5 (57.7) Range: [4.3-216.7]	<.001
**FTND**	-	0.0 (0.2)	0.3 (0.9)	.047
**Smoking initiation age (years)**	-	14.3 (1.4)	14.6 (1.1)	.315
**E-cigarette smoking in last 30 days**	0.0 (0.2)	0.4 (1.5)	0.0 (0.2)	.203
**Cannabis smoking in last 30 days**	0.0 (0.2)	0.4 (1.5)	0.2 (0.8)	.268
**Mean alcohol intake per week (glasses)**	11.0 (5.5)	15.6 (8.1)	17.6 (10.0)	<.001^b^

Abbreviations: FTND, Fagerström Test for Nicotine Dependence; MRSS-POS, Modified Reasons for Smoking Scale – pleasure of smoking scale; PDS, Pubertal Developmental Scale; SRHI, Self-Report Habit Index.

Missing data was imputed with the mean or the modus (one value for educational level in controls and one value for educational level in experimental smoking adolescents).

a controls < regular smoking adolescents (*P* = .011).

b controls < experimental & regular smoking.

### Imaging results

#### Whole brain analyses

One sample *t*-test of the whole group showed that smoking-related stimuli, compared to neutral stimuli, activated expected areas in the occipital and frontal cortices and amygdala/hippocampus, bilaterally (*P*_FWE_ <.05; Supplementary Figure 1, Supplementary Table 2), while smoking-related stimuli, compared to neutral stimuli, triggered lower activation in the parietal and temporal cortices (*P*_FWE_ <.05; Supplementary Figure 2, Supplementary Table 3).

Whole-brain analyses revealed no significant cluster differences between all groups (*P*_FWE_ >.05).

#### ROI analyses

**Figure 1:**
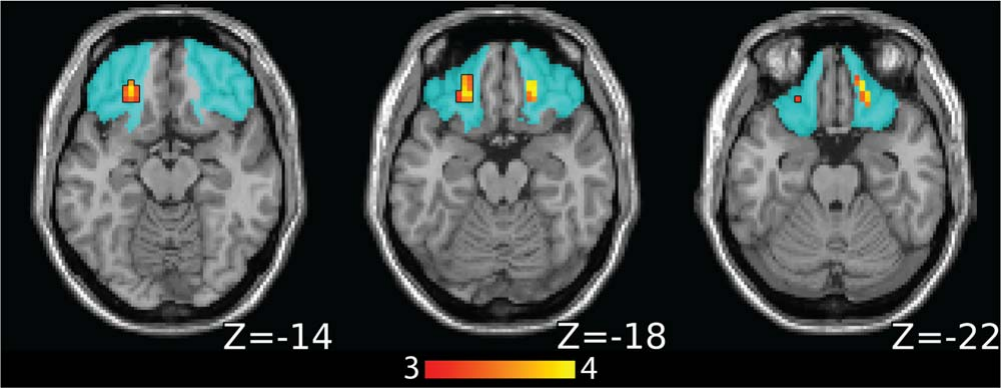
Small brain volume correction analysis of regular smoking adolescents > controls contrast. OFC (blue) with significant cluster in left OFC (*P* = .040) and trend-level cluster in the right OFC (*P*_FWE_ = .059). Color bar indicates T-value of specific voxel.

Region of interest analyses with small volume correction for the complete bilateral OFC revealed a significant higher activity cluster in the left OFC in regular smoking adolescents compared to controls (*P*_FWE_ = .040; Figure 1). The right OFC cluster was trend-level non-significant (*P*_FWE_ = .059; Figure. 1). There was no significant difference between experimental and regular smoking adolescents, or between controls and experimental smoking adolescents.

Exploratory sensitivity analyses with additional covariates for the number of e-cigarettes and cannabis smoking in the last 30 days, and glasses of alcohol per week showed similar group differences patterns in the OFC. As expected, when including more covariates, the power decreased, resulting in slightly increased *P*-values (*P*_FWE_ = .196, see Supplementary Table 4). We executed an exploratory ROI regression analysis between smoking-related cue activity and glasses of alcohol consumed per week. This resulted in insignificant clusters in the bilateral OFC (*P*_FWE_ >.176, see Supplementary Table 5). As the pattern of brain response remained the same and given the low amount of both e-cigarette and cannabis smoking in the sample (see Table 1), as well as no relation between OFC activity and alcohol intake (Supplementary Table 5), we consider our results robust against potential confounding by e-cigarette and cannabis use, and alcohol intake.

The MFG, dlPFC, and striatum revealed no significant differences between groups. Exploratory ROI analyses for ventral and dorsal striatum separately revealed no significant differences between controls and regular smoking adolescents.

#### Relationship smoking cue-reactivity, habitual smoking, pleasure of smoking, or number of cigarettes per week

We found no correlations between brain activity in the OFC and MRSS-POS scores (pleasure) or SRHI scores (habit). Exploratory whole-brain analyses, including SRHI and MRSS-POS scores, also revealed no significant correlations. Additional exploratory ROI analysis between smoking-cue reactivity and number of cigarettes smoked per month revealed no significant correlations.

## Discussion

To address neurobiological differences in cue-reactivity during the various onset phases of nicotine use, we investigated smoking cue-related brain activity in non-smoking, experimental smoking (<1/week), and regular smoking adolescents (>1/week). Whole-brain analyses showed no significant differences in smoking cue-reactivity between the groups. ROI analyses showed a significant higher smoking cue-related activity in the OFC in regular smoking adolescents compared to controls, but not in the striatum, dlPFC, or MFG. We found no significant dose–response relationships between smoking cue-related activity in the OFC and pleasure of smoking, habitual smoking, or number of cigarettes per month.

The current findings of unaltered whole-brain cue-reactivity during the onset phase of nicotine use in adolescents might be explained by the very low tendency for nicotine dependence in our sample, including regular smoking adolescents. This is in line with the observed positive correlation between smoking cue-related brain activity and nicotine dependency measured with the FTND.^34^ Our results suggest that significant brain changes in substance-related cue-reactivity may only emerge in individuals with higher substance dependence levels, as seen in adults with TUD^11^^,^^12^ and adults with various levels of alcohol use.^15–17^

Significant increases in smoking cue-related activity of the OFC were found in regular smoking adolescents compared to controls using a ROI small volume correction approach. This increased OFC activity is in line with the increased cue-reactivity observed in adults with TUD compared to healthy controls.^11^^,^^12^ This suggests there might be some initial similarities between non-dependent regular smoking adolescents and adults with TUD with regard to cue-reactivity. The OFC is related to reward-related behavior via reward-association learning of visual, olfactory, or palatable stimuli,^35^^,^^36^ and has been implicated in cue-reactivity in SUD.^11^ It is hypothesized that disruption of OFC function contributes to compulsive behavior associated with SUD.^37^ More specifically, activity of the OFC seems to be generally decreased in patients with SUD, while OFC activity is increased when they see drug-related cues or are (anticipate) taking substances, directing behavior toward compulsive substance use. The OFC might thus be a key structure involved in the development of SUD, including TUD, already contributing in the early stages of developing TUD.

We found no dose–response relationships between smoking cue-related activity in the OFC and pleasure of smoking or habitual smoking. Although activity of the OFC has been linked to subjective pleasure for different stimuli, such as olfactory, visual, and/or textual stimuli,^38^ our sample has relatively low levels of pleasure of smoking, with little variation within the smoking sample. Previous literature suggests that hypersensitivity for drug-related cues occurs mostly in the absence of pleasure of use.^39^ It remains to be determined if a more diverse range of pleasure of smoking will provide a relation with smoking-related cue activity of the OFC.

Taken together, our findings underscore the relative early phase and low levels of nicotine use in our sample. With the gradual development of more excessive smoking habits, both behavioral consequences, being habit formation, compulsivity, and cue-reactivity, as well as neurobiological changes, may become more apparent. Still, we observed increased smoking cue-related activation specifically in the OFC, suggesting that the OFC may already be involved in the onset phase of developing TUD. Additionally, current literature linked smoking cue-related activity in the OFC to craving in adult populations with TUD.^34^^,^^40^ Though craving is unlikely to occur in the very early phases of substance use, including nicotine use, future research should investigate whether the found differences in smoking cue-related activity of the OFC between non-smoking and regular smoking adolescents are related to craving in non-dependent samples as well as patients with TUD.

Based on our observational findings, no causal inferences can be made. It is unclear if the observed altered cue-reactivity in the OFC is caused by regular (weekly) smoking or represents a predisposition to develop more regular smoking behavior. Animal studies provide the opportunity for a controlled longitudinal design to determine the causal relation between the amount of smoking and cue-reactivity in the brain. To our knowledge, such a study has not yet been executed. Yet, several longitudinal studies show neurobiological alterations developing during substance use. For example, Muller Herde et al.^41^ showed that continuous nicotine consumption decreased glutamate receptor density compared to baseline in rats, and Jedema et al.^42^ showed in rhesus macaques that 12 months of chronic cocaine use altered gray matter density, which could be partly reversed with abstinence. These studies suggest a causal path from substance use to brain alterations, which can be reversed with abstinence.

Several studies suggest that pre-existing alterations in cue-reactivity and reward-related brain function may predispose to excessive substance use and SUD. For instance, a study focusing on the development of drinking behavior in moderately drinking young adults (17-22 years) showed that baseline cue-reactivity in the ventromedial prefrontal cortex, OFC, caudate nucleus, insula, and pre-central gyrus was associated with increased heavy drinking a year later,^18^ but not with heavy drinking in the month prior to the measurements. Similarly, Morales et al.^43^ showed that heightened activation of the reward circuit during decision making predicted an earlier onset of binge drinking. Both studies suggest that altered OFC function may predispose to developing problematic drinking. Furthermore, there are some longitudinal studies showing baseline neurobiological changes related to substance use later in life. For example, thinner prefrontal region cortices and larger hippocampal volumes at baseline were associated with earlier substance use initiation at follow-up.^44^ Additionally, negative relations between baseline fractional anisotropy of white matter tracts such as the corpus callosum and corona radiata and adolescent alcohol misuse at follow-up have also been reported.^45^ Furthermore, a lower OFC-to-amygdala volume ratio was linked to an increased change to develop SUD later on, regardless of familial risk.^46^ However, no such longitudinal study exists for the development of smoking behavior. Although these studies focused on different types of SUDs, they illustrate how preexisting OFC differences can precede substance use, which may also be true for nicotine. Yet, causality between smoking and OFC changes is not established.

It should be noted, though, that development of smoking behavior is influenced by many more factors than altered brain function. Examples include being male, being practically educated,^3^ having close friends that smoke [OR: 1.99-3.23], having parents that smoke [OR: 1.56-2.28],^47^ social media use for >5 hours/day [adjusted OR: 1.41-2.59] or being in the lowest 20% household income range [adjusted OR: 1.25-2.56].^48^ Future research should investigate the development of smoking behavior in longitudinal studies to determine the temporal relationship between smoking behavior and altered OFC cue-reactivity to understand this reactivity as a predisposition or the outcome of smoking behavior, in relation to other vulnerability factors for initiating and maintaining smoking behavior and developing TUD.

Although smoking prevalence is decreasing worldwide,^3^ previous studies showed an increase in vaping nicotine over the last couple of years, especially in young adults (18-35 years old).^49^ Similar to smoking, vaping cues increase self-reported craving for both vaping and smoking.^50^^,^^51^ Furthermore, vaping cues, as well as smoking cues, increase the activity in cue-reactivity related areas (medial prefrontal cortex and posterior cingulate cortex) in (non-smoking) vaping-dependent individuals.^33^ Exploratory analyses with e-cigarettes and cannabis smoking, and glasses of alcohol per week, did not change our results substantially. However, our sample included few participants using e-cigarettes or smoking cannabis. Future studies should further investigate differences and similarities in cue-reactivity among different forms of smoking behavior, including e-cigarettes/vaping and cannabis smoking.

Currently, there are universal preventive strategies used to lower the prevalence of tobacco use. For example, smoke-free environments, such as schools or sports, bans on tobacco and nicotine product advertisements, and raising taxes to increase the price of tobacco products,^52^ as well as strategies used for populations at risk, such as learning better coping mechanisms.^53^ These preventive strategies are of importance, as preventing the development of TUD, instead of treating, will reduce global burden of smoking worldwide. Our findings show that smoke-free environments are important as they reduce options to experience smoking cue-reactivity and accompanied OFC activation in smoking adolescents.

### Strengths and limitations

To the best of our knowledge, this is the first study that investigated how neurobiological differences in cue-reactivity develop in adolescents in the onset phase of smoking behavior by comparing non-smoking, experimental smoking, and regular smoking adolescents. In contrast to many other studies, the included sample consisted for a large proportion of practically educated adolescents, making our findings important for generalization in the light of current educational demographics of smoking populations.^3^ However, there are several limitations of this study that require further discussion. First, we included a sample that consisted mostly of female participants (~70%), which may limit the generalizability of findings to male adolescents. Previous research has shown that smoking behavior is more prevalent in males compared to females, in both adolescents and adults,^3^ and that smoking initiation age is lower for males than females.^54^ One study by Wetherill et al.^55^ state that smoking cues, compared to neutral cues, increased activity in the OFC in both tobacco-dependent males and females, but additionally activated ventral striatum/pallidum in males only. The sex-dependent smoking-cue-related activation of the brain might explain our lack of significant results in the striatum in our predominantly female sample. However, our small male sample size prohibited to test this hypothesis. Therefore, future studies should aim for a more balanced sex representation. Secondly, the current study only included participants without TUD, while to fully understand the development from non-using to full TUD, adolescents with TUD should be included as well. Thirdly, the current study has an observational design. This makes it impossible to determine any temporal relationships between the number of cigarettes smoked and the cue-reactivity within the OFC.

### Conclusion

In summary, our results indicate that the alterations in smoking cue-related activation of the OFC in TUD may already exist during the onset phase of nicotine use, with weekly smoking behavior during adolescence. This suggests that the transition to altered smoking cue-reactivity as seen in TUD might start in the OFC before it spreads to other areas like the MFG, dlPFC, or striatum. Future studies on smoking cue-reactivity in smoking adolescents should include participants with a more established smoking pattern, including those with TUD, to provide more insight into different stages of smoking behavior, dependence, and TUD and their neurobiology. Additionally, larger and longitudinal studies are needed to provide deeper insights into the development of cue-reactivity in both adolescents and adults.

## Supplementary Material

Supplementary_2_ntag045(1)

## Data Availability

The meta data underlying this article are available at OSF via https://osf.io/6jw9p/.
