# Supplementary material for: The role of the orbitofrontal cortex in smoking cue-reactivity in onset phase of smoking behavior, a fMRI study in adolescents
Source: Nicotine Tob Res. 2026 Feb 23;28(7):1238–46. doi: 10.1093/ntr/ntag045 (PMC13286646; doi:10.1093/ntr/ntag045)
Supplement: Supplementary_2_ntag045(1) [file supplementary_2_ntag045(1).docx]

# Supplementary

## Supplementary Materials

### Learning effects on smoking cue-related brain activation

We assessed task learning with a paired sample t-test of all participants who were assigned the same group (e.g., experimental or regular smoking adolescents) at baseline and follow-up (n=35). We found no task learning effects between baseline and follow-up scans (P_FWE_ > 0.05). Therefore, we did not include measurement number as covariate in our analyses.

### Reaction time

For each participant separately, mean (standard deviation) reaction time in milliseconds and accuracy percentages were calculated for romantic, neutral and smoking cues. Two mixed model ANOVAs were used to test differences between groups (control, experimental, and regular smoking adolescents) between type of stimulus (neutral, romantic, and smoking), and the interaction of group and type of stimulus, on (1) reaction time and (2) accuracy. No covariates were added.

Mixed models ANOVA analysis for accuracy percentage revealed no effect of group, type of stimuli, or group * type of stimuli interaction (F[8,387]= 0.733, p=0.6562). Additionally, ANOVA analysis for mean reaction time was also non-significant (F[8,387]=1.944, p=0.052).

**Supplementary Table 1.** Mean (standard deviation) reaction time in milliseconds and accuracy percentage range.

|  | Romantic | | Neutral | | Smoke | |
| --- | --- | --- | --- | --- | --- | --- |
|  | RT mean(SD) | Accuracy(%) | RT mean(SD) | Accuracy(%) | RT mean(SD) | Accuracy(%) |
| Controls* | 546.1(123.9) | 84.4-100 | 565.5(171.2) | 84.4-100 | 569.6(166.2) | 84.4-100 |
| Experimental | 473.3(101.0) | 87.5-100 | 489.3(121.1) | 93.8-100 | 504.9(142.3) | 87.5-100 |
| Regular | 479.9(172.1) | 93.8-100 | 484.1(149.8) | 93.8-100 | 506.7(171.1) | 87.5-100 |
| * One control is missing from analyses | | | | | | |

### General smoking cue-related activity


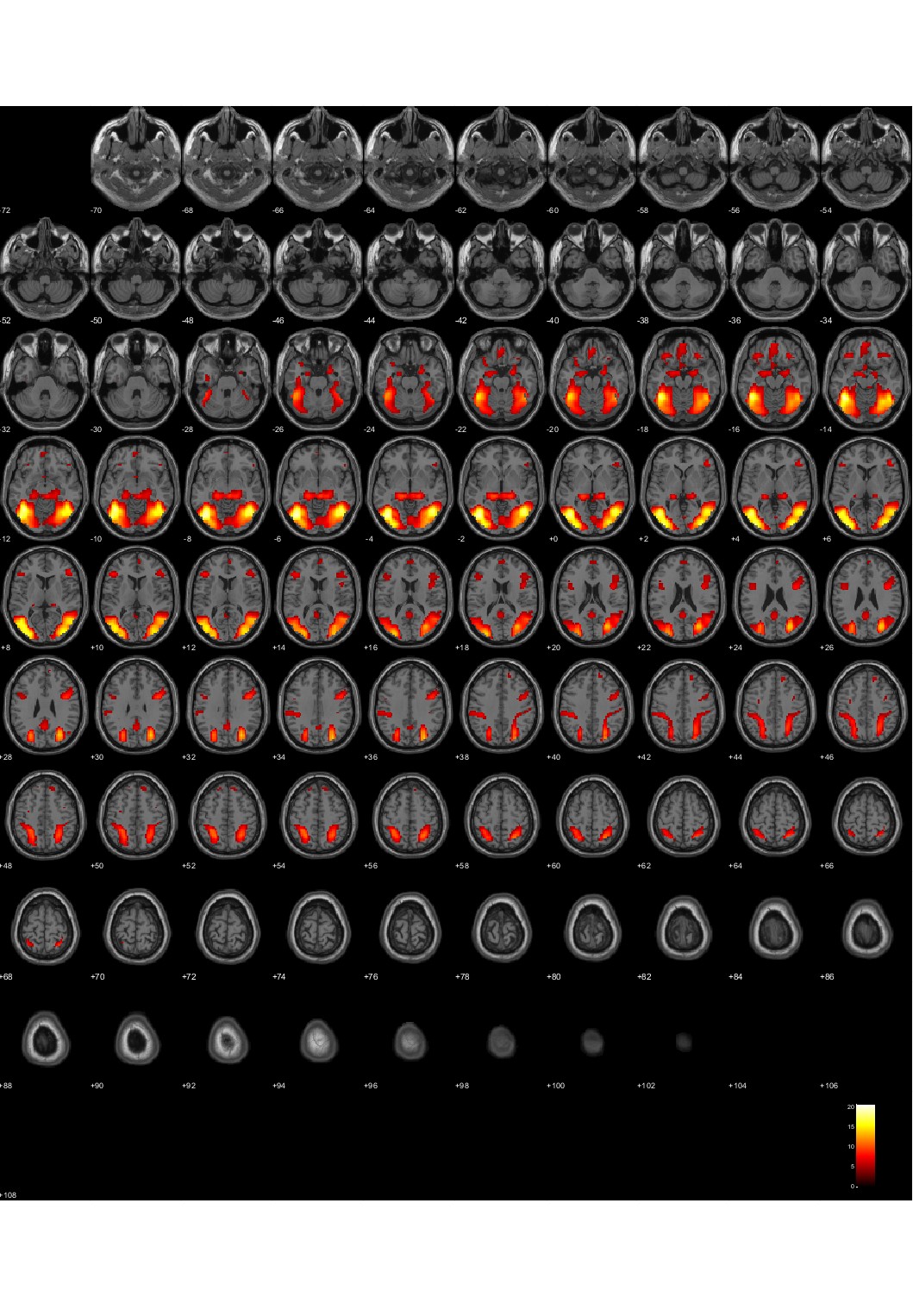


**Supplementary Figure 1.** Group effect (n=133) of active cue-reactivity for the smoking stimuli > neutral stimuli contrast (P_FWE_ <0.05; axial view). Colour bar indicates T-value of specific voxel.

**Supplementary table 2.** Whole brain analysis of the smoking stimuli > neutral stimuli contrast.

|  |  |  | MNI coordinates | | |
| --- | --- | --- | --- | --- | --- |
| Brain area | P_FWE_ | Cluster size (K_E_) | X | Y | Z |
| Left OFC (Fo6) | 0.000 | 4654 | -41 | 53 | -14 |
| Right middle precentral sulcus | 0.000 | 339 | 43 | 4 | 32 |
| Left IFS (IFS1) | 0.000 | 56 | -45 | 32 | 11 |
| Left amygdala | 0.000 | 203 | -20 | -4 | -18 |
| Left posterior orbital gyrus | 0.000 | 57 | -34 | 32 | -14 |
| Right subparietal sulcus | 0.000 | 103 | 1 | -56 | 28 |
| Left inferior precentral sulcus | 0.000 | 48 | -41 | 4 | 25 |
| Right superior frontal gyrus | 0.000 | 33 | 19 | 42 | 42 |
| Right DLPFC | 0.000 | 32 | 5 | 53 | 18 |


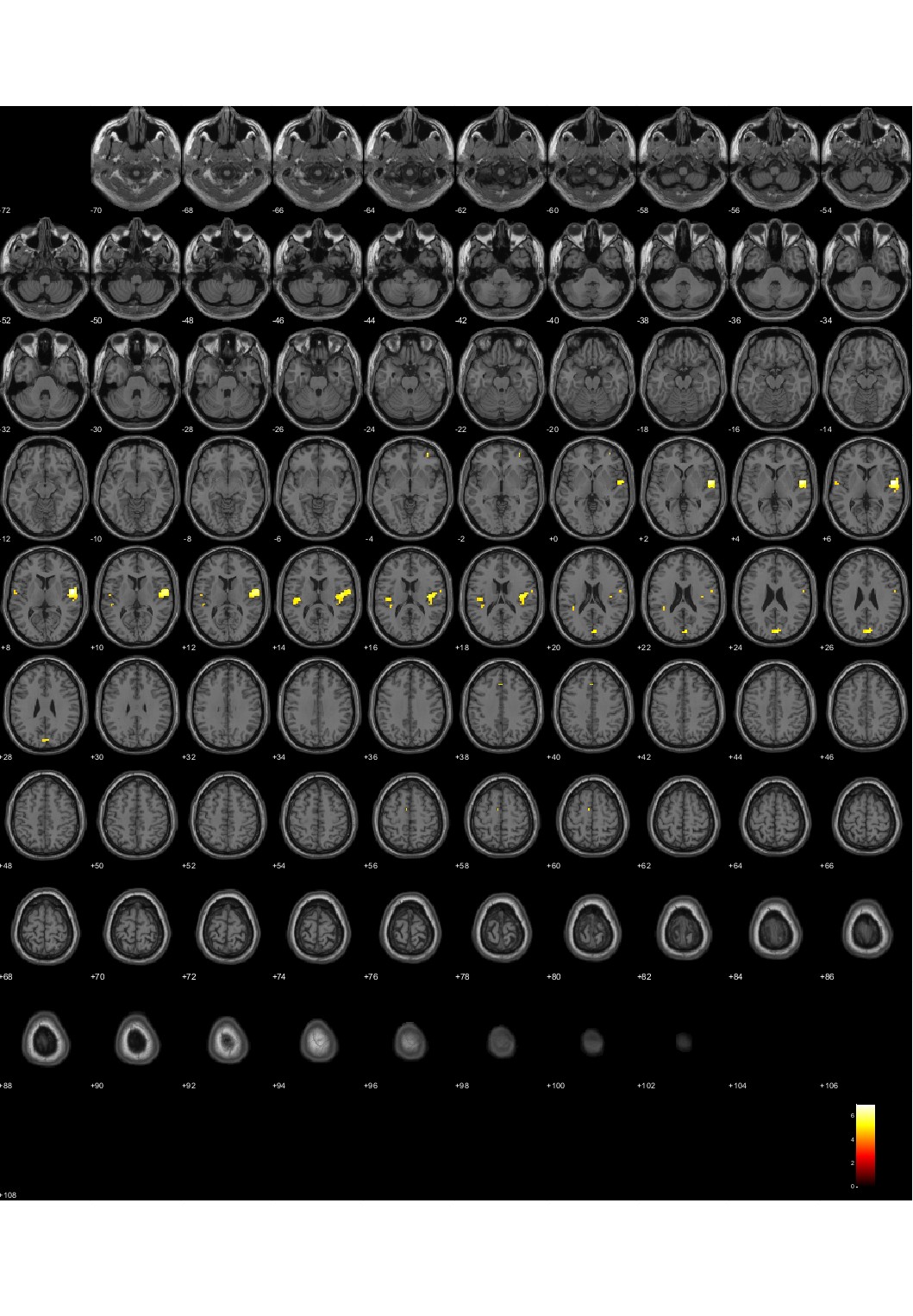


**Supplementary Figure 2.** Group effect (n=133) of active cue-reactivity for the smoking stimuli < neutral stimuli contrast (P_FWE_ <0.05; axial view). Colour bar indicates T-value of specific voxel.

**Supplementary Table 3.** Whole brain analysis of the smoking stimuli < neutral stimuli contrast.

|  |  |  | MNI coordinates | | |
| --- | --- | --- | --- | --- | --- |
| Brain area | P_FWE_ | Cluster size (K_E_) | X | Y | Z |
| Right Heschl’s gyrus | 0.000 | 128 | 57 | -4 | 7 |
| Right superior cuneus | 0.000 | 21 | 5 | -84 | 25 |
| Left middle lateral fissure | 0.000 | 22 | -41 | -21 | 14 |
| Right frontomarginal sulcus | 0.013 | 3 | 36 | 53 | -4 |
| Left posterior lateral fissure | 0.007 | 5 | -38 | -35 | 21 |
| Left medial mid-posterior superior frontal gyrus | 0.018 | 2 | -6 | -4 | 60 |
| Middle paracingulate gyrus | 0.018 | 2 | 1 | 28 | 39 |
| Left central opercular cortex | 0.013 | 3 | -55 | -7 | 7 |
| Left Heschl’s gyrus | 0.026 | 1 | -52 | -11 | 11 |

### Sensitivity analyses with number of e-cigarette and cannabis smoking in the last 30 days, and alcohol use in the last week

ROI analyses with small volume correction for the complete bilateral OFC, including additional covariates for number of e-cigarettes and cannabis smoking in the last 30 days revealed a non-significant higher activity cluster in the OFC in regular smokers compared to controls (Supplementary Table 4).

**Supplementary Table 4.** OFC ROI analysis of the smoking stimuli < neutral stimuli contrast.

|  |  |  | MNI coordinates | | |
| --- | --- | --- | --- | --- | --- |
| Brain area | P_FWE_ | Cluster size (K_E_) | X | Y | Z |
| Left OFC | 0.196 | 1 | -24 | 35 | -18 |

Additional exploratory OFC ROI regression analysis between smoking stimuli < neutral stimuli contrast acivity and alcohol intake per week showed no significant clusters

**Supplementary Table 5.** OFC ROI regression analysis of the smoking stimuli < neutral stimuli contrast with alcohol intake.

|  |  |  | MNI coordinates | | |
| --- | --- | --- | --- | --- | --- |
| Brain area | P_FWE_ | Cluster size (K_E_) | X | Y | Z |
| Left Frontal-to-Temporal-II | 0.201 | 2 | -20 | 7 | -21 |
| Right OFC (Fo1) | 0.236 | 1 | 15 | 42 | -21 |
| Left Frontal-to-Temporal-II / Left OFC (Fo2) | 0.236 | 1 | -13 | 14 | -21 |
| Left OFC (Fo3) | 0.176 | 3 | -24 | 46 | -14 |
| Right OFC (Fo3) | 0.201 | 2 | 22 | 35 | -18 |
| Left OF (Fo2) | 0.236 | 1 | -17 | 18 | -18 |
